# Efficacy of Longdan Xiegan Decoction on the Treatment of Eczema: A Systematic Review and Meta-Analysis

**DOI:** 10.1155/2021/8836117

**Published:** 2021-02-18

**Authors:** Ziteng Hu, Lidong Gao, Chengxian Li, Alberto Cucco, Shang Wang, Weiling Yuan, Fan Zhang, Shuai Kang, Min Wang

**Affiliations:** Tianjin University of Traditional Chinese Medicine, Tianjin 301617, China

## Abstract

**Background:**

Longdan Xiegan decoction (LDXGD) has been widely used in the treatment of eczema. In recent years, randomized controlled trials (RCTs) of LDXGD for the treatment of eczema have gradually increased. Most of the results show that LDXGD is effective in treating eczema. However, whether these conclusions are reliable or not requires meta-analysis.

**Objective:**

This study aimed to systematically evaluate the clinical efficacy of LDXGD in the treatment of eczema.

**Materials and Methods:**

Seven electronic databases, including PubMed, Excerpta Medica Database (EMBASE), Cochrane Library, Chinese Biomedical Literature on Disc (CBM), China National Knowledge Infrastructure (CNKI), WanFang, and Chinese Science and Technology Periodical Database (VIP) were systematically searched from their inception until January 2021. Risk of bias was assessed using criteria from the Cochrane Collaboration and meta-analysis was conducted on the screened literature data using Review Manage (RevMan 5.3). Then, to assess the quality of evidence, the GRADE criteria was adopted.

**Results:**

14 RCTs with 1080 participants were identified. Meta-analysis indicated that compared with western medicine (WM), the cure rate and the total effective rate of LDXGD in treating eczema were higher. Meanwhile, the recurrence rate and the levels of interleukin-6 (IL-6), interleukin-8 (IL-8), and tumor necrosis factor (TNF-*α*) after treatment were lower. The adverse reaction was reported in 5 out of 14 studies without significant statistical difference. According to GRADE criteria, the quality of evidence was low for all outcomes except for the cure rate (moderate-quality evidence) and the total effective rate (moderate-quality evidence).

**Conclusion:**

The clinical efficacy of LDXGD in the treatment of eczema was more effective compared with the one of conventional WM alone. However, due to the limitation of the quality of the included studies, additional studies are required to further confirm these results.

## 1. Introduction

Eczema is a skin inflammatory disease caused by a variety of internal and external factors, including immune factors, genetic factors, endocrine changes, environmental factors, and infectious factors [1]. It is characterized by pleomorphic skin lesions and recurrent attacks, and it is easy to become chronic [2]. Papules, blisters, exudation, erosion, and pruritus are common symptoms accompanying eczema [3], which significantly affect the patients' physical health and life quality. The pathogenesis of eczema is not completely clear. Studies have shown that the main cause is represented by internal factors, such as abnormal immune function and skin barrier dysfunction [4]. In China, the prevalence of eczema has been on the rise, accounting for about 15%∼20% in the dermatology department [5]. Antihistamine and glucocorticoid are the main measures to treat eczema. However, the long-term use of these drugs may lead to resistance, dependence, and a series of adverse reactions [6].

Longdan Xiegan decoction (LDXGD) is originated from Wang Ang's Prescriptions and composed of 10 herbs, including Longdancao (*Radix Gentianae*), Huangqin (*Radix Scutellariae*), Zhizi (*Fructus Gardeniae*), Zexie (*Rhizoma Alismatis*), Mutong (*Caulis Akebiae*), Cheqianzi (*Semen Plantaginis*), Shengdihuang (*Radix Rehmanniae*), Danggui (*Radix Angelicae Sinensis*), Chaihu (*Radix Bupleuri*), and Gancao (*Radix Glycyrrhizae*) [7]. It is widely used in the treatment of eczema. LDXGD can improve the CD4^+^T cell proportion in the body, while reducing the CD8^+^T cell proportion [8], and it improves the cellular immune regulation function after increasing the spleen index, which may be one of the main mechanisms in the treatment of eczema [9]. In recent years, there has been a gradual increase in the number of RCTs in the treatment of eczema with the LDXGD, and most of the results have shown that LDXGD is effective in treating eczema. However, whether these conclusions are reliable or not requires meta-analysis. Therefore, this paper adopted the method of systematic evaluation to compare the efficacy of LDXGD with WM in the treatment of eczema, to provide a real and objective basis for the clinical application of LDXGD in the treatment of eczema.

## 2. Methods

This study was conducted following the Preferred Reporting Items for Systematic Reviews and Meta-Analyses (PRISMA) [10].

### 2.1. Search Strategy

Seven electronic databases, including PubMed (https://http://www.ncbi.nlm.nih.gov/pubmed/), EMBASE (https://www.embase.com), Cochrane Library (https://www.cochranelibrary.com), CBM (http://www.sinomed.ac.cn/), CNKI (http://www.cnki.net/), WanFang (http://www.wanfangdata.com.cn/), and VIP (http://www.cqvip.com/) were systematically searched from their inception until January 2021. The following search terms were used and varied depending on which database was searched: “eczema”, “eczemas”, “dermatitis”, “eczematous”, “dermatitides”, “eczematous dermatitides”, “eczematous dermatitis”, “longdan xiegan”, “longdanxiegan”, “long dan xie gan”, “randomized controlled trial”, “randomized”, and “placebo”.

### 2.2. Inclusion Criteria

1. Type of participants: research involved patients with any type of eczema.
2. Type of study: only RCTs that assessed the efficacy of LDXGD for the treatment of eczema were eligible.
3. Type of intervention: LDXGD must be included in the herbal formula used in the experimental group. There were no restrictions on the drug dosage, frequency, or treatment time. The control group was treated with WM, including cetirizine, loratadine, ebastine, and azelastine.
4. Type of results: the efficacy of LDXGD on the treatment of eczema was evaluated through the cure rate and the total effective rate. Secondary outcomes included the recurrence rate and the levels of IL-6, IL-8, and TNF-*α* after treatment. Safety was evaluated through adverse events.

### 2.3. Exclusion Criteria

If the above-described conditions were not met, the literature is to be excluded. Besides, the following conditions should also be excluded:

1. Duplicate publications.
2. Animal experiments, mechanisms, studies, reviews, experience, and case reports.
3. Not available full text of the literature.
4. Whenever in the experimental group, LDXGD was used as adjuvant therapy or contained other traditional Chinese medicine formulation.
5. Whenever the control group contained LDXGD.

### 2.4. Study Selection

Two reviewers (Fan Zhang and Shuai Kang) separately searched the aforementioned databases and listed the titles of all articles. According to the inclusion criteria, by looking through the title and abstract, they excluded papers that were not eligible. Next, they screened the contents of the unclear articles further. If there were some overlaps or repetitions in the article, only the latest information was included. Through discussions with the corresponding authors of the study, the dispute about the selection of documents was resolved.

### 2.5. Data Extraction

According to the inclusion and exclusion criteria, two reviewers (Fan Zhang and Shuai Kang) independently screened the literature, extracted the data, and carried out cross-checking. When differences arose, a third party (Weiling Yuan) was involved in discussion or consultation, to assist in judgment. The extracted data included the first author, publication time, basic data of the study subjects, the sample size of the experimental group and control group, specific details of the intervention measures, outcome indicators, and data [11].

### 2.6. Quality Assessment

Two reviewers (Lidong Gao and Chengxian Li) independently evaluated the methodological quality of these trials according to Cochran's Systematic Review Handbook risk assessment tool. The risk of bias includes 7 items: random sequence generation (selection bias), allocation concealment (selection bias), blinding of participants and personnel (performance bias), blinding of outcome assessment (detection bias), incomplete outcome data (attrition bias), selective reporting (reporting bias), and other bias. Differences among the reviewers were resolved by a discussion [12].

### 2.7. Data Analysis

RevMan 5.3 statistical software was used for statistical analysis. Relative risk (RR) and 95% confidence interval (CI) were used for the binomial variables. The mean difference (MD) and 95% CI were used when the continuous variables were the same unit of measurement, and the standard mean difference (SMD) and 95% CI were used when different units of measurement were used. Heterogeneity was judged on the basis of the results of *I*^*2*^-test. The fixed-effect model was adopted when *I*^2^ < 50%. *I*^2^ > 50% indicated that the heterogeneity of interstudy was significant, so the reasons for heterogeneity must be analyzed. Firstly, it was checked whether the original data and the method of data extraction were correct. Secondly, if the heterogeneity attributed to race, course, dose, random method, allocation concealment, and so on, subgroup analysis could be used. Sensitivity analysis could also be performed to explore the causes of heterogeneity. If the cause of heterogeneity still was not to be explained, the random effect model could be used. Sensitivity analysis was conducted on the stability and reliability of meta-analysis results. Finally, a meta-analysis was given up if the combined results of the study had no clinical significance. Inverted funnel plots were used to determine potential publication bias when more than 10 studies were included in the meta-analysis. *P* < 0.05 was considered statistically significant. GRADE criteria were used to access the strength of the evidence to make the results more credible.

## 3. Results

### 3.1. Search Results

**Figure 1:**
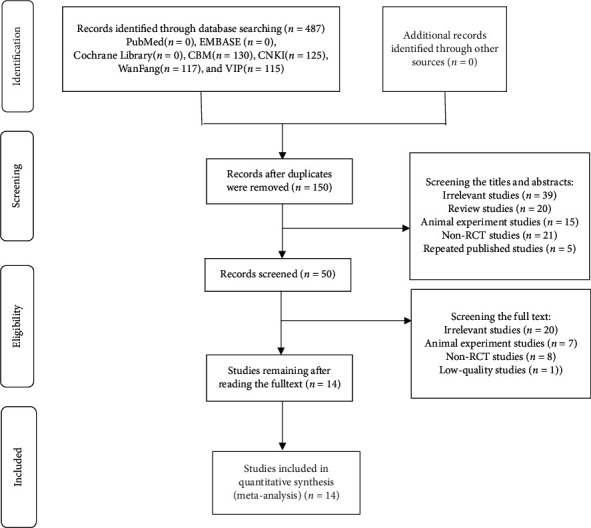
Inclusion process and results of the relevant articles.

A total of 487 studies were retrieved from the seven electronic databases (PubMed (*n* = 0), EMBASE (*n* = 0), Cochrane Library (*n* = 0), CBM (*n* = 130), CNKI (*n* = 125), WanFang (*n* = 117), and VIP (*n* = 115)) and other sources. After removing the duplicates, 150 records remained. By screening the titles and abstracts, 100 records were excluded (irrelevant studies (*n* = 39), review studies (*n* = 20), animal experiment studies (*n* = 15), non-RCT studies (*n* = 21), and repeated published studies (*n* = 5)). By reading the full text, 36 records were removed (irrelevant studies (*n* = 20), animal experiment studies (*n* = 7), non-RCT studies (*n* = 8), and low-quality studies (*n* = 1)). Finally, 14 studies were included (see Figure 1).

### 3.2. Study Characteristics

A total of 14 RCTs involving 1080 patients were included, published from 2009 to 2019. All studies were published in China. Sample sizes ranged from 50 to 125, with significant differences in the course of disease and age. The intervention of the experimental group included LDXGD alone or combined with WM, while the control group was based on WM monotherapy, including cetirizine, loratadine, ebastine, and azelastine. The course of treatment ranged from 10 to 21 days. Five studies [13–17] reported the incidence of adverse reactions. The basic characteristics of the 1080 patients were consistent, and no significant differences were found before the intervention (see Table 1).

### 3.3. Methodological Quality Assessment

**Figure 2:**
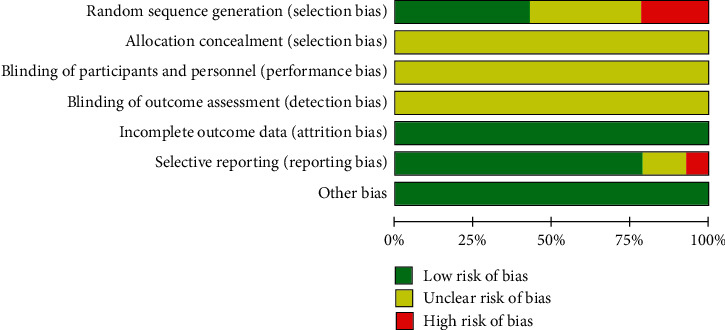
Risk of bias graph of the 14 articles.

**Figure 3:**
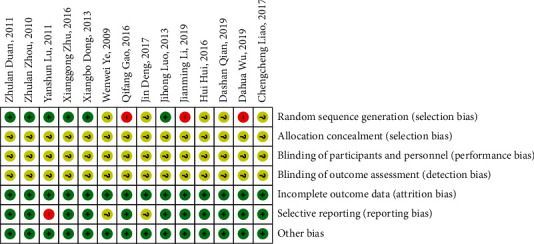
Risk of bias summary graph of the 14 articles.

Although randomization was announced in all of the included trials, 6 studies [16, 18, 20, 21, 25, 26] used a random number table or described the procedure of randomization; accordingly, the risk of bias on the domain was judged as “low risk.” Three studies [15, 22, 23] had “high risk” because the random sequence was generated based on the clinic number or the date of visit. The remaining studies [13, 14, 17, 19, 24] only reported “random” without a specific method and evaluated were as “unclear risk.” Allocation concealment and blinding of participants and outcome assessment were not mentioned in any of the studies and assessed as “unclear risk.” All studies [13–26] indicated that the outcome data were complete and were assessed as “low risk.” In selective reporting, 11 studies [14–16, 18–24, 26] were assessed as “low risk” because the published reports contained all the expected results, 2 studies [13, 17] were assessed as “unclear risk” due to lack of sufficient information, and 1 study [25] that reported only the curative effect calculated based on eczema area and severity index (EASI) without reporting the raw data was assessed as “high risk.” We evaluated other biases according to the comparability of baseline data in the trials, like gender, age, and duration between groups; lastly, all studies were judged as “low risk” (see Figures 2 and 3).

### 3.4. Primary Outcomes

The curative-effect standard of eczema referred to “Guiding Principles of Clinical Research of Traditional Chinese Medicine.” Standard of cure: all skin lesions and itching symptoms disappeared. The treatment efficiency reached 100%. Standard of significantly effective: most of the skin lesions disappeared, and pruritus symptoms were significantly reduced. The treatment efficiency was more than 70%. Standard of effective: skin lesions partially disappear, and pruritus symptoms were improved. The treatment efficiency was more than 30%. Standard of invalidity: skin lesions had not subsided significantly, and pruritus had not improved or worsened. Or the treatment efficiency had not reached the effective standard. The total effective rate was the sum of the cure rate, significant effective rate, and effective rate.

#### 3.4.1. Cure Rate

**Figure 4:**
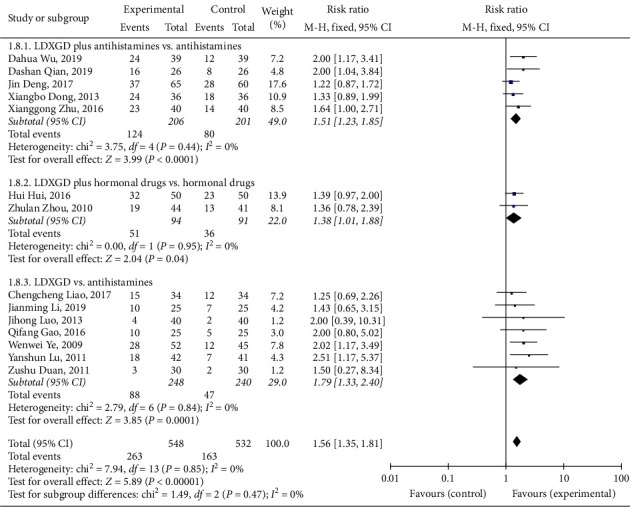
Forest plot and meta-analysis of the cure rate.

A total of 14 [13–26] studies reported the cure rate, and meta-analysis showed that LDXGD was better at improving the cure rate of eczema (RR = 1.56, 95% CI (1.35, 1.81), *P* < 0.00001, heterogeneity *I*^2^ = 0%, *P*=0.85, Figure 4). Five studies compared LDXGD plus antihistamines with antihistamines, and there was a significant difference between them (RR = 1.51, 95% CI (1.23, 1.85), *P* < 0.0001, heterogeneity *I*^2^ = 0%, *P*=0.44, Figure 4) [13–16, 18]. Two studies compared LDXGD plus hormonal drugs with hormonal drugs, and there was a significant difference between them (RR = 1.38, 95% CI (1.01, 1.88), *P*=0.04, heterogeneity *I*^2^ = 0%, *P*=0.95, Figure 4) [19, 20]. Seven studies compared LDXGD with antihistamines, and there was a significant difference between them (RR = 1.79, 95% CI (1.33, 2.40), *P*=0.0001, heterogeneity *I*^*2*^ = 0%, *P*=0.84, Figure 4) [17, 21–26].

#### 3.4.2. Total Effective Rate

**Figure 5:**
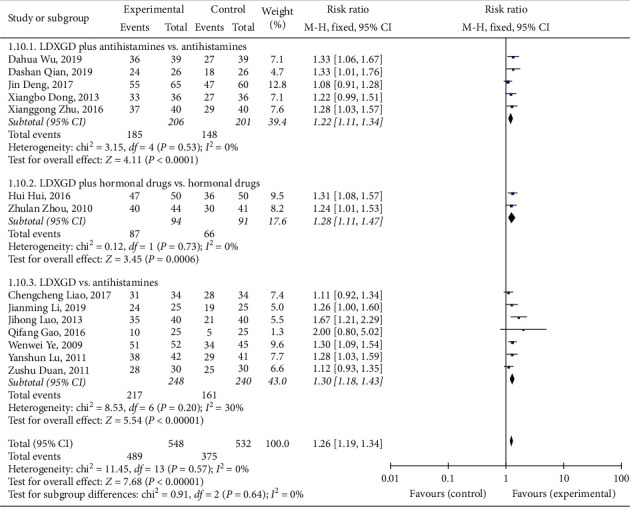
Forest plot and meta-analysis of the total effective rate.

A total of 14 [13–26] studies reported the total effective rate, and meta-analysis showed that LDXGD was better at improving the total effective rate of eczema (RR = 1.26, 95% CI (1.19, 1.34), *P* < 0.00001, heterogeneity *I*^2^ = 0%, *P*=0.57, Figure 5). Five studies compared LDXGD plus antihistamines with antihistamines, and there was a significant difference between them (RR = 1.22, 95% CI (1.11, 1.34), *P* < 0.0001, heterogeneity *I*^2^ = 0%, *P*=0.53, Figure 5) [13–16, 18]. Two studies compared LDXGD plus hormonal drugs with hormonal drugs, and there was a significant difference between them (RR = 1.28, 95% CI (1.11, 1.47), *P*=0.0006, heterogeneity *I*^2^ = 0%, *P*=0.73, Figure 5) [19, 20]. Seven studies compared LDXGD with antihistamines, and there was a significant difference between them (RR = 1.30, 95% CI (1.18, 1.43), *P* < 0.00001, heterogeneity *I*^2^ = 30%, *P*=0.20, Figure 5) [17, 21–26].

### 3.5. Secondary Outcomes

#### 3.5.1. Recurrence Rate

**Figure 6:**
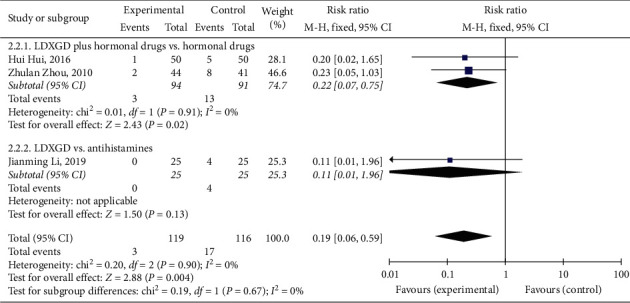
Forest plot and meta-analysis of the recurrence rate.

A total of 3 [19, 20, 23] studies reported the recurrence rate. The results showed that the recurrence rate of LDXGD plus hormonal drugs was lower than that of hormonal drugs alone (RR = 0.22, 95% CI (0.07, 0.75), *P*=0.02, heterogeneity *I*^2^ = 0%, *P*=0.91, Figure 6) [19, 20]. There was no statistically significant difference between the LDXGD alone group and the antihistamines group (RR = 0.11, 95% CI (0.01, 1.96), *P*=0.13, Figure 6) [23].

#### 3.5.2. Levels of IL-6, IL-8, and TNF-*α* after Treatment

A total of 3 [14–16] studies reported the levels of IL-6, IL-8, and TNF-*α* after treatment. The results showed that the levels of IL-6 (SMD = −1.61, 95% CI (−1.91, −1.30), *P* < 0.00001, heterogeneity *I*^2^ = 0%, *P*=0.49), IL-8(SMD = −1.68, 95% CI (−1.99, −1.38), *P* < 0.00001, heterogeneity *I*^2^ = 13%, *P*=0.31) and TNF-*α* (SMD = −1.68, 95% CI (−1.98, −1.37), *P* < 0.00001, heterogeneity *I*^2^ = 0%, *P*=0.59) after treatment of LDXGD plus antihistamines were lower than the one of antihistamines alone, and the difference was statistically significant (see Table 2).

### 3.6. Adverse Reaction Rate

**Figure 7:**
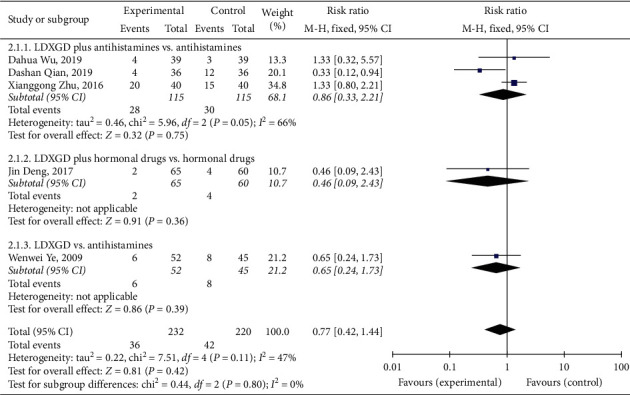
Forest plot and meta-analysis of the adverse reaction rate.

The main adverse reactions included abdominal pain, diarrhea, nausea, dry mouth, fatigue, dizziness, and lethargy. A total of 5 [13–17] studies reported the adverse reaction rate. Meta-analysis showed that there was no statistically significant difference between the experimental group and the control group (RR = 0.77, 95% CI (0.42, 1.44), *P*=0.42, heterogeneity *I*^2^ = 47%, *P*=0.11, Figure 7).

### 3.7. Sensitivity Analysis

Sensitivity analysis showed that all single studies could not change the final outcomes, which meant that this meta-analysis had good stability.

### 3.8. Publication Bias

**Figure 8:**
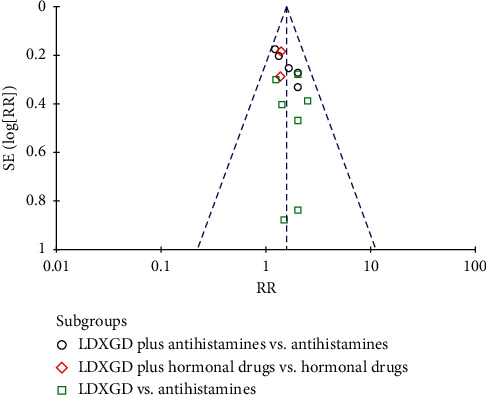
Funnel plot of the cure rate.

**Figure 9:**
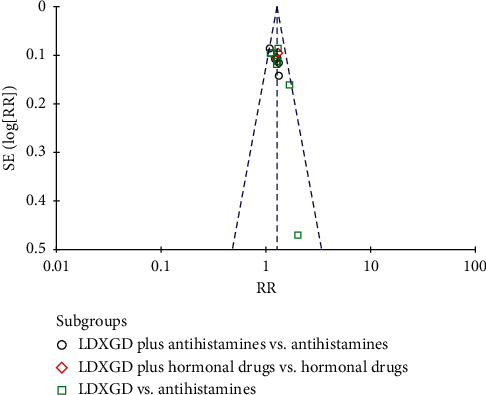
Funnel plot of the total effective rate.

Publication bias was analyzed by funnel plots of the total effective rate and the cure rate. The funnel plots showed incomplete symmetry between right and left, which may be caused by factors such as a small number of selected studies, low quality, unpublished negative results, or small sample effect (see Figures 8 and 9).

### 3.9. Evidence Grading Results

The risk of bias, inconsistency, indirectness, imprecision, and publication bias of each outcome indicator were analyzed and summarized. GRADE evaluation was conducted for each outcome indicator, among which the cure rate and the total effective rate were moderate-quality evidence, and the recurrence rates and posttreatment levels of IL-6, IL-8, and TNF-*α* were low-quality evidence (see Table 3). The main reason for the downgrade of the primary outcomes was that the original research had a certain degree of publication bias, which affected the authenticity of the research results. And the reasons for the downgrade of the second result evidence were mainly due to a certain degree of selection bias in the original study and the small number of included studies.

## 4. Discussion

### 4.1. Summary of Evidence

In summary, compared with WM used alone, LDXGD used alone or combined with WM had a higher cure rate (RR = 1.56, 95% CI (1.35, 1.81), *P* < 0.00001) and total effective rate (RR = 1.26, 95% CI (1.19, 1.34), *P* < 0.00001) in treating eczema, and lower levels of IL-6 (SMD = −1.61, 95% CI (−1.91, −1.30), *P* < 0.00001), IL-8 (SMD = −1.68, 95% CI (−1.99, −1.38), *P* < 0.00001), and TNF-*α* (SMD = −1.68, 95% CI (−1.98, −1.37), *P* < 0.00001) after treatment. The recurrence rate of LDXGD combined with WM was lower than that of WM used alone (RR = 0.22, 95% CI (0.07, 0.75), *P*=0.02), and there was no statistically significant difference between LDXGD used alone and WM used alone (RR = 0.11, 95% CI (0.01, 1.96), *P*=0.13). As well, there was also no significant difference in adverse reaction rate between the experimental group and the control group (RR = 0.77, 95% CI (0.42, 1.44), *P*=0.42). According to the GRADE criteria, the cure rate and total effective rate were moderate-quality evidence, while the recurrence rate and the levels of IL-6, IL-8, and TNF-*α* after treatment were low-quality evidence.

### 4.2. Limitations

There are several limitations in our primary studies as well. Firstly, the methodological quality of the 14 RCTs included in this study was not high. Some RCTs did not elaborate on the specific randomization method, and all RCTs did not elaborate on allocation concealment or whether the blind method was used, which may affect the conclusions of this study. Secondly, the RCTs included in this study were all published studies, and due to the limitation of retrieval resources, part of the gray literature was not available, so potential publication bias could not be excluded. Finally, ITT analysis was not performed on the final data in all RCTs, which was likely to cause a loss of follow-up bias. Thus, the conclusions of this meta-analysis need to be provided with more reliable evidence-based medical evidence by future multicenter, large-sample, and high-quality randomized controlled clinical trials.

### 4.3. Phytochemical Investigations and Pharmacological Activities

LDXGD is composed of 10 herbs and can improve the CD4^+^T cell proportion in the body while reducing the CD8^+^T cell proportion [8]. It also improves the cellular immune regulation function after increasing the spleen index, which may be one of the main mechanisms in the treatment of eczema [9]. The immune regulation, anti-inflammatory, free radical scavenging, and antioxidant effects of LDXGD all play an important role in the treatment of eczema [7].

#### 4.3.1. Longdancao (Radix Gentianae)

Longdancao contains numerous kinds of chemical components, mainly including iridoid glycosides, alkaloids, and flavonoids [27], among which flavonoids have distinctive anti-inflammatory effects [28]. Research suggests Longdancao can reduce dermal edema, telangiectasia, and inflammatory cell infiltration and achieve the same effect as glucocorticoids in suppressing the inflammatory response to eczema in some aspects [29].

#### 4.3.2. Huangqin (Radix Scutellariae)

So far, over 40 compounds have been isolated and identified from Huangqin, including flavonoids, terpenoids, volatile oils, and polysaccharides [30]. Flavonoids inhibit the expression of cyclooxygenase-2 (COX-2) gene to prevent the transcription factor CCAAT/enhancer binding protein (C/EBP) from binding to deoxyribonucleic acid (DNA), thus inhibiting the metabolism of arachidonic acid producing anti-inflammatory effects [31]. Wogonoside could decrease the production of inflammatory mediators nitric oxide (NO) and prostaglandin E2 (PGE2) and inhibit the release of proinflammatory cytokines such as TNF-*α* and IL-6 [32].

#### 4.3.3. Zhizi (Fructus Gardeniae)

Zhizi contains iridoid glycosides, organic acid esters, saffron glycosides, and flavonoids [33]. Geniposide can significantly reduce the expression levels of cytokines such as interleukin 4 (IL-4), IL-6, and TNF-*α* in the blood of Nc/Nga inflamed mice and obviously relieves the inflammatory symptoms of mice [34]. Fang's experiments also proved that geniposide has a certain anti-inflammatory effect [35].

#### 4.3.4. Chaihu (Radix Bupleuri)

Dual regulation of Chaihu to adenylate cyclase can enhance the immunity of mice [36]. Bupleurum polysaccharides and bupleurum saponin, the active components of Chaihu, can inhibit the inflammatory response, and the mechanism of action is related to stimulating the adrenal gland and promoting the function of the adrenal cortex system [37, 38].

#### 4.3.5. Danggui (Radix Angelicae Sinensis)

Modern research indicates that phthalides, organic acids and their esters, and polysaccharides are the main chemical components related to the bioactivities and pharmacological properties of Danggui [39]. Ferulic acid and isoferulic acid contribute to the anti-inflammatory activity of Danggui [40, 41]. Angelica polysaccharide has the function of promoting phagocytic cell phagocytosis and promoting lymphocyte transformation and proliferation [42].

#### 4.3.6. Shengdihuang (Radix Rehmanniae)

Shengdihuang contains iridoid glycosides, glycosides, amino acids, and other active ingredients [43]. Zhao's experimental results show that rehmannia glutinosa polysaccharides can significantly increase the spleen index of mice and enhance the phagocytic function of mononuclear macrophages [44]. Wang's research results indicate that Rehmannia glutinosa polysaccharides can clearly increase the synthesis of lymphocyte DNA and protein and can obviously enhance the production of interleukin-2 (IL-2) in active lymphocytes and enhance immune function [45]. Liang's experiment also shows that Shengdihuang has the effect of regulating immunity [46].

#### 4.3.7. Gancao (Radix Glycyrrhizae)

Modern pharmacological research shows that Gancao contains total flavonoids, glycyrrhizic acid, glycyrrhetinic acid, triterpenoids, glycyrrhizin, and other ingredients [47]. Guo's research found that both isooligonucleotides and naringenin, the effective components of Gancao, can enhance the immunosuppressive effect of Treg cells [48]. Yang's research confirmed that total flavonoids in Gancao downregulate the inflammatory factors nitric oxide synthase (iNOS), IL-6 and COX-2, and downregulate and activate iNOS and COX-2 in macrophages, and upregulate the expression of peroxisome proliferator-activated receptor *γ* (PPAR-*γ*) messenger ribonucleic acid (mRNA) to achieve anti-inflammatory effects [49].

#### 4.3.8. Zexie (Rhizoma Alismatis)

The chemical components of Zexie include triterpenes, sesquiterpenes, and diterpenes [50]. The alcohol extract, water extract, and various monomer components of Zexie have anti-inflammatory effects [51].

#### 4.3.9. Mutong (Caulis Akebiae)

The main components of Mutong are triterpenes and their saponins [52]. Acanthopanax saponins A, hederagenin, and oleanolic acid obtained from Mutong have anti-inflammatory effects, and the anti-inflammatory effect of hederagenin is stronger than the other two compounds [53].

#### 4.3.10. Cheqianzi (Semen Plantaginis)

The chemical components of Cheqianzi mainly include polysaccharides, phenethyl alcohol glycosides, iridoids, flavonoids, alkaloids, triterpenes, and sterols [54]. Zhang's research found that Psyllium extract can reduce the permeability of rat skin and abdominal capillaries and the permeability of red blood cell membranes, indicating that Psyllium has anti-inflammatory activity [55]. Chen's research showed the effect of Psyllium macrophage polysaccharide (PL-PS) on the production of nitric oxide in leukemia cells in mouse macrophage (RAW264.7 cells) and found that PL-PS is a macrophage immunomodulatory substance.

## 5. Conclusion

The results indicated that the clinical efficacy of LDXGD used alone or combined with WM was superior to that of WM alone in treating eczema, but it was not possible to draw a definite conclusion as for the safety of LDXGD. The curative effect of LDXGD on eczema is certain according to the moderate-quality evidence assessed through GRADE, while other outcomes were uncertain based on current studies. Thus, further research is needed to find more convincing proof.

## Figures and Tables

**Table 1:** The general characteristics of the 14 trials.

Study	Sample size (T/C)	Course of the disease (mean or range) (T/C)	Age (mean or range) (T/C)	Intervention		Duration of use	Outcome
				T	C		
Jin Deng, 2017 [12]	65/60	NR	44.2 ± 5.3/42.8 ± 4.6	LDXGD + ebastine	Ebastine	10d	1, 2, 4
Xiangbo Dong, 2013 [13]	36/36	NR	NR	LDXGD + ebastine	Ebastine	14d	1, 2
Hui Hui, 2016 [18]	50/50	NR	NR	LDXGD + compound econazole nitrate cream	Compound econazole nitrate cream	15d	1, 2, 3
Dashan Qian, 2019 [19]	26/26	(3.25 ± 1.13/4.23 ± 1.11) m	35.31 ± 10.24/36.25 ± 10.16	LDXGD + azelastine	Azelastine	10d	1, 2, 4, 5, 6, 7
Dahua Wu, 2019 [14]	39/39	NR	NR	LDXGD + cetirizine	Cetirizine	21d	1, 2, 4, 5, 6, 7
Zhulan Zhou, 2010 [15]	44/41	NR	38.95 ± 15.36/39.31 ± 15.71	LDXGD + qumixin cream	Qumixin cream	21d	1, 2, 3
Xianggong Zhu, 2016 [20]	40/40	(5.5 ± 2.3/5.3 ± 2.1) m	44.1 ± 4.8/43.5 ± 4.7	LDXGD + cetirizine	Cetirizine	10d	1, 2, 4, 5, 6, 7
Zushu Duan, 2011 [16]	30/30	(5.15 ± 3.76/6.74 ± 4.47) d	37.54 ± 9.36/35.13 ± 12.91	LDXGD	Cetirizine	14d	1, 2
Qifang Gao, 2016 [21]	25/25	(9.03 ± 0.96/8.64 ± 0.81) d	41.09 ± 8.03/40.63 ± 7.65	LDXGD	Loratadine	NR	1, 2
Jianming Li, 2019 [22]	25/25	(2.1 ± 0.60/2.10 ± 0.57) y	36.6 ± 2.17/36.78 ± 2.25	LDXGD	Cetirizine	21d	1, 2, 3
Chengcheng Liao, 2017 [23]	34/34	(1.53 ± 0.53/1.32 ± 0.64) d	34.67 ± 12.75/35.21 ± 13.25	LDXGD	Loratadine	14d	1, 2
Yanshun Lu, 2011 [24]	42/41	(5.39 ± 1.22/5.27 ± 2.13) d	36.25 ± 4.38/35.94 ± 6.03	LDXGD	Levocetirizine	21d	1, 2
Jihong Luo, 2013 [25]	40/40	(2.6 ± 1.1/2.8 ± 1.3) y	64.2 ± 5.4/65.3 ± 4.8	LDXGD	Cetirizine	21d	1, 2
Wenwei Ye, 2009 [26]	52/45	112/113 d	36.6/36.9	LDXGD	Ebastine	20d	1, 2, 4

T: experimental group; C: control group; NR: no record; d: day; m: month; y: year; 1: cure rate; 2: total effective rate; 3: recurrence rate; 4: adverse reaction rate; 5: IL-6; 6: IL-8; and 7: TNF-*α*.

**Table 2:** Analysis of IL-6, IL-8, and TNF-*α* levels after treatment.

Outcomes	Number of included studies	Heterogeneity		Effect model	SMD (95%CI)	*P*
		*P*	*I* ^2^ (%)			
IL-6	3	0.49	0	Fixed-effects model	−1.61 [−1.91, −1.30]	<0.00001
IL-8	3	0.31	13	Fixed-effects model	−1.68 [−1.99, −1.38]	<0.00001
TNF-*α*	3	0.59	0	Fixed-effects model	−1.68 [−1.98, −1.37]	<0.00001

**Table 3:** Evidence grading of the outcomes.

Interventions for [condition] in [population]							
Outcomes	Intervention and comparison intervention	Illustrative comparative risks^*∗*^ (95% CI)		Relative effect (95% CI)	Number of participants (studies)	Quality of the evidence (GRADE)	Comments
		Assumed risk	Corresponding risk				
		**With comparator**	**With intervention**				
*Primary outcomes*							
	Cure rate	**Study population**		**RR 1.56** (1.35 to 1.81)	1080 (14 studies)	⊕ ⊕ ⊕ ⊝ **moderate**	
		**306 per 1000**	**478 per 1000** (414 to 555)				
		**Moderate**					
		**308 per 1000**	**480 per 1000** (416 to 557)				

	Total effective rate	**Study population**		**RR 1.26** (1.19 to 1.34)	1080 (14 studies)	⊕ ⊕ ⊕ ⊝ **moderate**	
		**705 per 1000**	**888 per 1000** (839 to 945)				
		**Moderate**					
		**728 per 1000**	**917 per 1000** (866 to 976)				

*Secondary outcomes*							
	Recurrence rate	**Study population**		**RR 0.19** (0.06 to 0.59)	235 (3 studies)	⊕ ⊕ ⊝⊝ **low**	
		**147 per 1000**	**28 per 1000** (9 to 86)				
		**Moderate**					
		**160 per 1000**	**30 per 1000** (10 to 94)				

	IL-6		The mean LDXGD combined with WM in the intervention groups was **1.61 standard deviations lower** (1.91 to 1.3 lower)		226 (3 studies)	⊕ ⊕ ⊝⊝ **low**	SMD −1.61 (−1.91 to −1.3)

	IL-8		The mean LDXGD combined with WM in the intervention groups was **1.68 standard deviations lower** (1.99 to 1.38 lower)		226 (3 studies)	⊕ ⊕ ⊝⊝ **low**	SMD −1.68 (−1.99 to −1.38)

	TNF-*α*		The mean LDXGD combined with WM in the intervention groups was **1.68 standard deviations lower** (1.98 to 1.37 lower)		226 (3 studies)	⊕ ⊕ ⊝⊝ **low**	SMD −1.68 (−1.98 to −1.37)

^*∗*^The basis for the assumed risk (e.g., the median control group risk across studies) is provided. The corresponding risk (and its 95% confidence interval) is based on the assumed risk in the comparison group and the relative effect of the intervention (and its 95% CI). CI: confidence interval; RR: relative risk; SMD: standard mean difference. GRADE working group grades of evidence: high quality: further research is very unlikely to change our confidence in the estimate of effect; moderate quality: further research is likely to have an important impact on our confidence in the estimate of effect and may change the estimate; low quality: further research is very likely to have an important impact on our confidence in the estimate of effect and is likely to change the estimate; very low quality: we are very uncertain about the estimate.

## Data Availability

The data supporting this systematic review and mate-analysis are from previously reported studies and datasets, which have been cited. The processed data are available in the supplementary files.

## Supplementary Materials

Supplementary Materials Table S1: search Strategy. Table S2: PRISMA Checklist.
