# Supplementary material for: Efficacy of Longdan Xiegan Decoction on the Treatment of Eczema: A Systematic Review and Meta-Analysis
Source: Evid Based Complement Alternat Med. 2021 Feb 18;2021:8836117. doi: 10.1155/2021/8836117 (PMC7906809; doi:10.1155/2021/8836117)
Supplement: Supplementary Materials — Table S1: search Strategy. Table S2: PRISMA Checklist. [file 8836117.f1.zip › 8836117.f1/Table S1.doc]

| Databases | Retrieval strategy |
| --- | --- |
| PubMed | #1 “eczema” [MeSH Terms] #2 “eczemas” [Title/Abstract]  #3 “dermatitis” [Title/Abstract]  #4 “eczematous” [Title/Abstract]  #5 “dermatitides” [Title/Abstract]  #6 “eczematous dermatitides” [Title/Abstract] #7 “eczematous dermatitis” [Title/Abstract]]  #8 (#1 OR #2 OR #3 OR #4 OR #5 OR #6 OR #7)  #9 “longdan xiegan” [Title/Abstract] #10 “longdanxiegan” [Title/Abstract] #11 “long dan xie gan” [Title/Abstract] #12 (#9 OR #10 OR #11)  #13 “randomized controlled trial” [Publication Type] OR “randomized” [Title/Abstract] OR “placebo” [Title/Abstract]  #14 (#8 AND #12 AND #13) |
| Embase | #1 ‘eczema’/exp #2 ‘eczemas’: ab, ti  #3 ‘dermatitis’: ab, ti #4 ‘eczematous’: ab, ti #5 ‘dermatitides’: ab, ti  #6 ‘eczematous dermatitides’: ab, ti #7 ‘eczematous dermatitis’ :ab, ti  #8 (#1 OR #2 OR #3 OR #4 OR #5 OR #6 OR #7)  #9 ‘longdan xiegan’: ab, ti #10 ‘longdanxiegan’: ab, ti #11 ‘long dan xie gan’: ab, ti #12 (#9 OR #10 OR #11)  #13 ‘randomized controlled trial’/exp |

|  | #14 (#8 AND #12 AND #13) |
| --- | --- |
| The Cochrane Library | #1 eczema*: ME  #2 dermatitis* OR eczematous dermatitis*  #3 longdan xiegan OR longdanxiegan OR long dan xie gan #4 (#1 OR #2 OR #3) |
| CBM  (Chinese) | #1 “湿疹”[加权：扩展]  #2 “湿疹”[常用字段：智能]OR“湿疮”[常用字段：智能]  #3 （#1）OR（#2）  #4 “龙胆泻肝汤”[常用字段：智能]  #5 （#3）AND（#4）  #6 “随机”[全部字段：智能]OR“盲法”[全部字段：智能]OR “安慰剂”[全部字段：智能] |
| CNKI  (Chinese) | #1 SU=（“湿疹”+“湿疮”）  #2 SU=（“龙胆泻肝汤”）  #3 FT=（“随机”+“盲法”+“安慰剂”）  #4 #1 AND #2 AND #3 |
| WanFang (Chinese) | #1 主题：“湿疹”+主题：“湿疮”  #2 主题：“龙胆泻肝汤”  #3 全部：“随机”+全部：“盲法”+全部：“安慰剂”  #4 #1 AND #2 AND #3 |
| VIP  (Chinese) | #1 题名或关键词：湿疹+湿疮  #2 题名或关键词：龙胆泻肝汤  #3 #1 AND #2 |
